# Internet Health Information–Seeking Trend of Urinary Incontinence in Mainland China: Infodemiology Study

**DOI:** 10.2196/55670

**Published:** 2025-06-23

**Authors:** Shuangquan Lin, Lingxing Duan, Xiongbing Lu, Haichao Chao, Xi Wen, Shanzun Wei

**Affiliations:** 1 Department of Urology, The Second Affiliated Hospital, Mingde Rd 1, Nanchang, 330000, China, 1 4588006725; 2 Laboratory of Urology, The Second Affiliated Hospital, Nanchang, China

**Keywords:** urinary incontinence, Baidu, Baidu index, Baidu encyclopedia, infodemiology, public interest, patients' concern

## Abstract

**Background:**

Urinary incontinence (UI) is a series of clinical episodes featuring involuntary urine leakage. UI affects people in terms of their physical, emotional, and cognitive functioning, and the negative perceptions and impact on patients are not fully understood. In addition, the true demand for the treatment of UI and related issues is yet to be revealed.

**Objective:**

The aim of this study is to examine the online search trend, user demand, and encyclopedia content quality related to UI on a national and regional scale on Baidu search, the major search engine in Mainland China.

**Methods:**

The Baidu Index was queried using UI-related terms for the period from January 2011 to August 2023. The search volume for each term was recorded to analyze the search trend and demographic distributions. For user interest, the demand graph data and trend data were collected and analyzed.

**Results:**

Three search topics were identified with the 18 available UI search keywords. The total Baidu search index for all UI topics was 11,472,745. The annual percent changes (APCs) for the topic Complaint were 1.7% (*P*<.05) from 2011-2021 and −7.9% (*P*<.05) from 2021-2023, and the average annual percent change (AAPC) was 0.1% (*P*<.05). For the topic Inquiry, the APCs were 16% (*P*<.05) from 2011 to 2016, −27.00% from 2016 to 2019, and 21.2% (*P*<.05) from 2019 to 2023, with an AAPC of 4.8%. Regarding the topic of Treatment, the APC was 20.3% from 2011-2018 (*P*<.05), −36.9% from 2018-2021 (*P*>.05), and 2.2% from 2021-2023, with a −0.4% overall AAPC. The age distribution of the population of each UI search topic inquiry shows that the search inquiries for each topic were mainly made by the population aged 30 to 39 years. People from the eastern part of China made up around 30% of each search query.

**Conclusions:**

Web-based searching for UI topics has been continuous and traceable since January 2011. Different categorized themes within the UI topic highlight specific demands from various populations, necessitating tailored responses. Although online platforms can offer answers, medical professionals’ involvement is crucial to avoid misdiagnosis and delayed treatment.

## Introduction

Urinary incontinence is a series of clinical episodes that involve involuntary urine leakage [1]. Based on the different mechanisms and symptomatic discrepancies, major phenotypes of UI are (1) stress UI (SUI), (2) urgency UI, (3) mixed UI, and (4) overflow incontinence, along with other defined categories [1,2]. The frequency of UI varies greatly depending on demography, gender, and definitional differences. The surveyed prevalence ranges from 30.3% to 45% in females and 3.5% to 6% in males [3]. In China, a survey of 518,465 people revealed the UI prevalence ranges from 37.1% to 45.1% [4]. In men over 60, the prevalence ranges from 35% to 56.3% [5,6]. Additionally, the yearly expense of UI ranges from US $7.6 billion (€7 billion) to US $66 billion; it is estimated to rise to $82.6 billion by 2020 [7,8].

UI affects people’s physical, emotional, and cognitive functioning [9]; the negative perceptions of urinary leakage problems negatively impact patients and affect their psychological status and social roles, impairing their overall quality of life and well-being [10]. It was reported that individuals with UI were more likely to conceal their distress and be hesitant to seek medical attention out of shame and stigma [11,12]. As a result, the known frequency of UI may be the tip of the iceberg [13,14].

Infodemiology, the study of infodemics, information distribution, and demand for online health information and misinformation, has been credited with revealing aspects of public health, social politics, and public perception, as well as guiding health professionals and patients to quality health information [15-17]. Infodemic research was carried out in the field of urology for both premature ejaculation and lower urinary tract symptoms, which were identified as the most widespread and concerning urologic issues [18,19]. Considering the possible adverse effects of UI on the well-being, quality of life, and social involvement of patients, the utilization of infodemiology as a metric may shed light on the unrecognized needs and demands of these populations through an alternative approach. Hence, this study aims to assess the online demand for UI information by analyzing search trends and inquiry distribution and user demand. Additionally, we aim to identify the most prominent issues pertaining to real-world geographic databases at present.

## Methods

### Keyword Selection and Data Retrieval

The UI keywords used for the search were determined by referring to the International Urogynecological Association and International Continence Society published terminology report [1,20]. Based on these reports, various UI-describing keywords for both genders and multiple specific conditions were listed to avoid omission. All synonyms or complex derivatives were screened and selected to minimize language habits derived from ambiguity and bias, as previously described [18,21]. All available UI keywords with established temporal search trend data were categorized into 3 topics according to their connotation and are listed in Multimedia Appendix 1.

As described in previous studies [18,21], three modules on the Baidu Index platform—(1) the searching trend, (2) user demand, and (3) the demographic portrait module—enabled the analysis of search demand by examining trends in popularity, topic-related concerns, and the geodemographic features of each topic. The Baidu search index (BSI) value is the key index that numerically scales the popularity of each search keyword. It is updated daily and is recorded at the national and subnational level [18,21]; therefore, further quantifying the BSI could allow for the analysis of the search trend, regional distribution, demographic preferences, and user interest. For each search keyword, the Baidu Index platform provides the BSI record for up to a decade, depending on the module, when required. Hence, the provincial and national trend data were collected from January 1, 2011, to August 31, 2023. In addition, the most recent within-range data from the geodemographic and search demand modules were collected [18,21].

The Baidu Index user demand platform delivers to users the most pertinent search results (“related terms”) for each keyword search on a weekly basis. These randomly generated related terms reflect users’ interests and queries. These data can be analyzed and ranked based on their frequency of appearance and popularity in searches.

To review the content from the Baidu Encyclopedia, the UI-related content on this popular science platform was also collected. All UI content was scored and sorted with a preliminary global quality scale assessment [22]. The number of visits, word count, and other essential information were documented.

### Data Analysis

For each UI topic, each public demand trend (the specific question proposed by the users) was presented with data sequentially plotted from BSI data in chronological order starting from January 1, 2011.

The overtime trend change for each domain was determined by the annual percent change (APC) model. The APC and average annual percentage change (AAPC) are statistical measures used in epidemiology and public health to quantify trends over time in rates or proportions of events, such as disease incidence or mortality rates. They are typically calculated using regression methods, such as joinpoint regression analysis.

APC measures the average rate of change in percentage terms over a single year. It provides insights into how a particular rate or proportion has been changing annually, that is, whether it has been increasing, decreasing, or remaining stable [23].

AAPC takes into account trends over multiple years, providing a summary measure of the average rate of change per year over a specified period. It gives a more comprehensive view of trends compared to APC, especially when trends are not linear. These models are designed to examine the change in popularity over a specified fixed interval of time [21,24,25]. *P*<.05 suggests that the trend is statistically significant.

For geographic disparities, the regional distribution of the demand for each UI term was computed annually based on provincial BSI values and sorted by the 7 Chinese administrative divisions as previously reported [21].

In the user demand section, the top 10 most frequently appearing related words for each search keyword were listed and sorted by their weekly popularity. This shows the frequency and popularity of each search-related issue on the Baidu index. Similar to previous findings, these user demand module data were retrieved and categorized into 13 categories to clarify the users’ main concerns and implied intentions [21]. These categories are (1) complaints, (2) inquiries, (3) treatment decisions, (4) health issues, (5) diagnoses, (6) hospitals and services, (7) symptom confirmation, (8) tests and examinations, (9) prognoses, (10) traditional Chinese medicine (TCM) complaints, (11) TCM inquiries, (12) TCM ingredients, and (13) TCM regimens, along with unrelated, random, or off-topic words (ie, irrelevant) [21].

### Statistical Analysis

The entire database was constructed using Excel 2019 (Microsoft Corp). The APC was calculated using the joinpoint regression model (version 4.7.0.0; Statistical Research and Applications Branch, National Cancer Institute). The intergroup differences were compared with the Fisher exact test or the Kruskal-Wallis test in applicable situations. A *P*<.05 was considered statistically significant. We used Prism 9 for macOS (version 9.5.0 [525]; GraphPad Software) to conduct statistical analysis and create figures.

### Ethical Considerations

We used publicly available, anonymized data that can be accessed without requiring any special permissions. Since the data are aggregated and publicly accessible, institutional review board approval was waived.

## Results

### The Data on Trends in UI Topics Are Currently Accessible

We summarized the total UI search requests on Baidu over the past 12 years with the described search keywords. Based on the analysis of these 18 keywords, three major topics were recognized for initial classification: (1) complaint with 1 search keyword, (2) inquiry with 3 search keywords, and (3) treatment with 4 search keywords (see Multimedia Appendix 1). The cumulative BSI of the UI keywords inside the trend module amounted to 11,472,745. Across all topics, the total value of BSI in 13 years was 8,174,724 in the complaint category, 1,671,387 in the inquiry category, and 1,626,634 in the treatment category.

**Figure 1.**
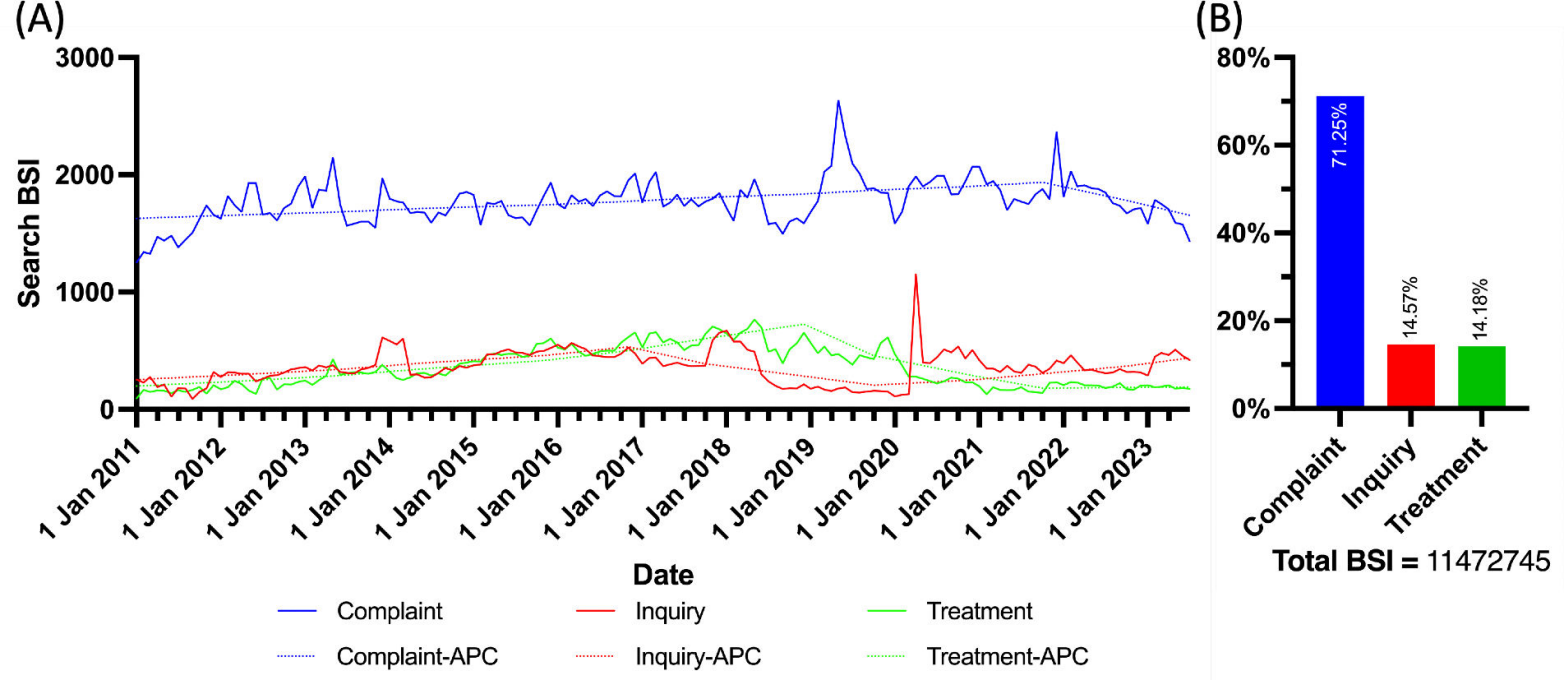
Online interest in urinary incontinence topics since 2011. (A) Search trend of topics about urinary incontinence. (B) Sum BSI proportion of each topic about urinary incontinence. APC: annual percentage change; BSI: Baidu search index.

Based on the annual average BSI count for each UI topic, it was observed that search requests in each topic underwent a comparable period of growth, culminating in a baseline returning trend by the conclusion of 2023. The particular APCs for the topic Complaint were 1.7% (*P*<.05) from 2011 to 2021 and −7.9% (*P*<.05) from 2021 to 2023, while AACP was 0.1% (*P*<.05). For the topic Inquiry, the APCs were 16.0% (*P*<.05) from 2011 to 2016, –27.00% from 2016 to 2019, and 21.2% (*P*<.05) from 2019 to 2023, with an AAPC of 4.8%. Regarding the topic Treatment, the APC was 20.3% from 2011 to 2018 (*P*<.05), –36.9% from 2018 to 2021 (*P*>.05), and 2.2% from 2021 to 2023, with an overall AAPC of −0.4%. The APC trend, the joint-point, and the AAPC over time were calculated and demonstrated in Figure 1.

### Geographic Disparities

**Figure 2.**
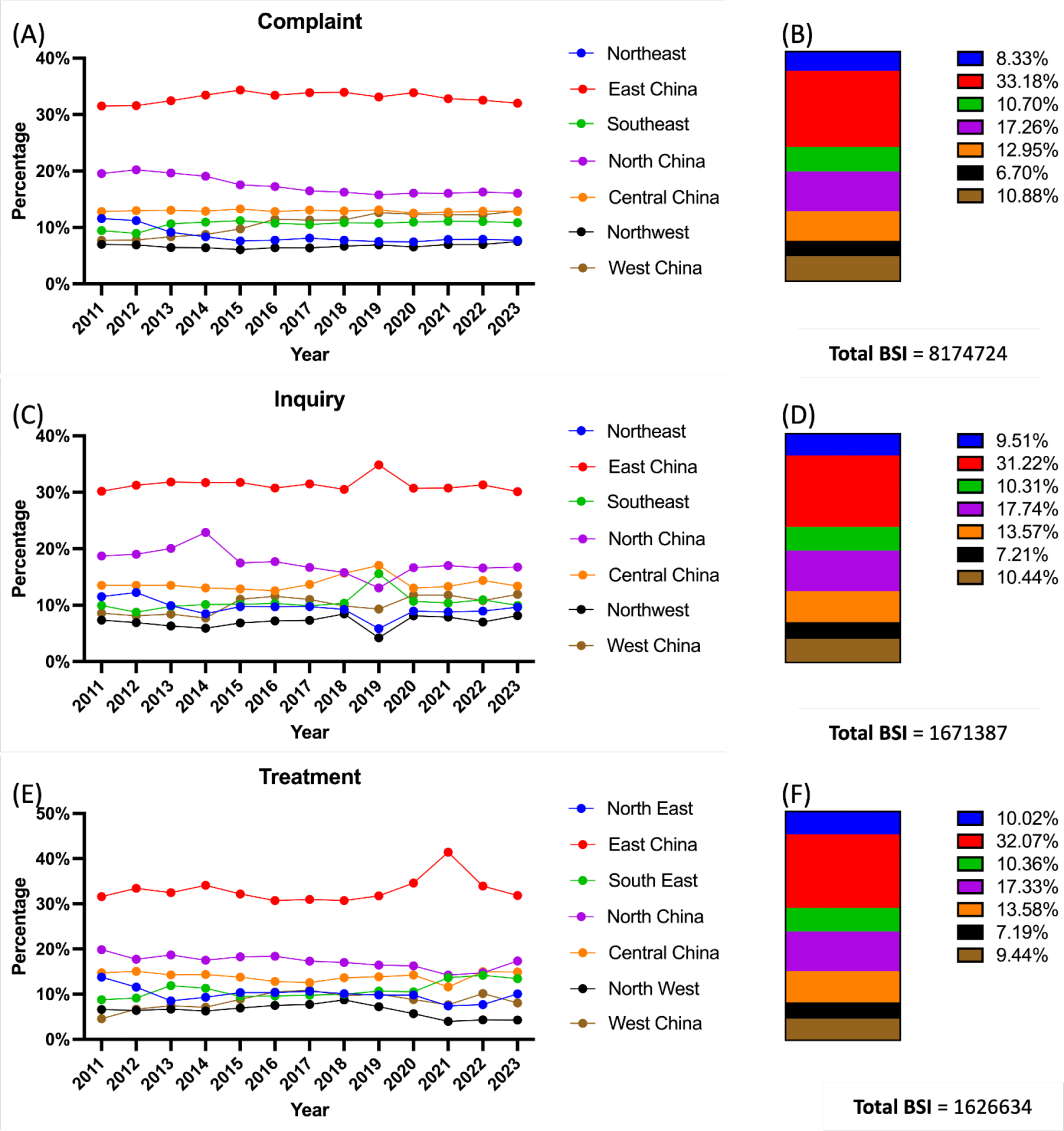
Online interest in urinary incontinence topics since 2011. (A) Annual trend of the BSI for each region for the topic of Complaint. (B) Total search rates of each area for the topic of Complaint. (C) Annual trend of the BSI for each region for the topic of Inquiry. (D) Total search rates for each area for the topic of Inquiry. (E) Annual trend of the BSI for each region for the topic of Treatment. (F) Total search rates for each area for the topic of Treatment. BSI: Baidu search index.

The 13-year regional BSI proportions for each UI topic accompanied by a valid search record are shown in Figure 2. East Chinese users comprised the majority of search query contributors, accounting for 33.18%, 31.22%, and 32.07% in the Complaint, Inquiry, and Treatment categories, respectively. The regions of North China and Central China were next in succession. The demand levels in the Northeast, West China, and South China were comparable, each contributing 12%. Northwest demand rates were last, contributing 6.7%, 7.21%, and 7.19% to each subject.

### Demographic Differences

**Figure 3.**
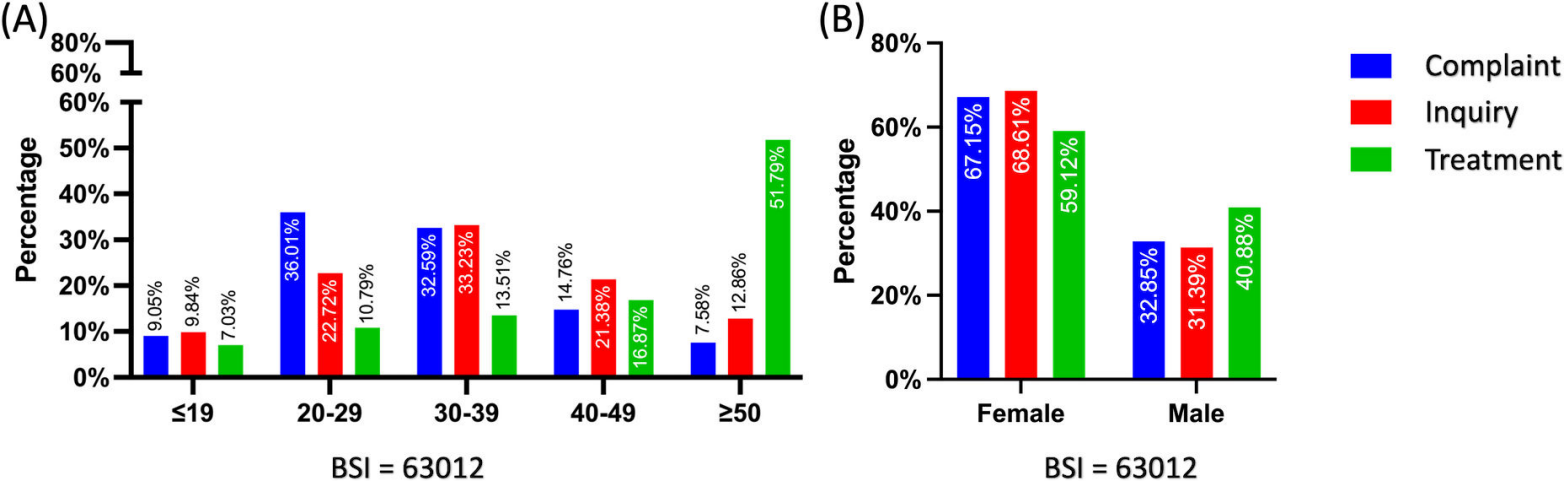
Population disparities in search preference for urinary incontinence topics. (A) Age disparities in search preference for urinary incontinence topics. (B) Gender disparities in search preference for urinary incontinence topics. BSI: Baidu search index.

From the topic-specific demographic distribution analysis, searches varied in age and gender distribution. Regarding the Complaint topic, users aged 20‐29 and 30-39 years made 36.01% and 32.59% of requests. Similarly, most of the requests in the Inquiry topic came from people aged 30-39 years (33.23%), followed by people aged 20-29 years (22.72%), and finally people aged 40-49 years (21.38%). In the Treatment topic, more than 51.79% of requests originated from users over 50, and this trend continued, with only 7.3% of requests coming from users under 19 years of age. In contrast, requests from this age group were below 10% in other topics. Regarding gender disparities, women made the majority of the total requests for each subject. Although the demand for Treatment was very low, around 60% of the requests were still made by female users (Figure 3).

**Figure 4.**
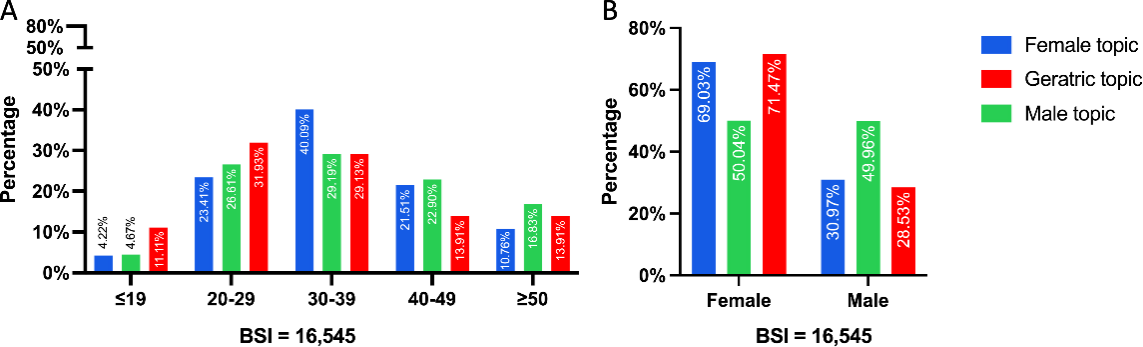
Population disparities in search preference for urinary incontinence topics specific to the female, male, and geriatric populations. (A) Age disparities in search preference for urinary incontinence topics specific to different populations. (B) Gender disparities in search preference for urinary incontinence topics specific to different populations. BSI: Baidu search index.

Furthermore, we discovered that some gender-specific and older adult–restricting information was indicated in particular search keywords. Herein, an additional topic in the female, geriatric, and male categories was sorted for examination. Most requests (40.09%) for the female topic came from the 30‐39 year old demographic. For the male issue, requests from the 30‐39 year old group ranked highest, but they also shared a similar proportion, at 20%‐30%, with the second- and third-highest groups. Regarding the geriatric subject, it was seen that a majority of the requests, namely 60%, were made by users aged between 20-29 and 30-39 years. Additionally, it was found that female users made 71.47% of these requests. In contrast, requests from the 40-49 and ≥50 age groups were just 13.91% and 13.91%, respectively (Figure 4).

### Keywords, Related Terms, and Search Frequency

**Figure 5.**
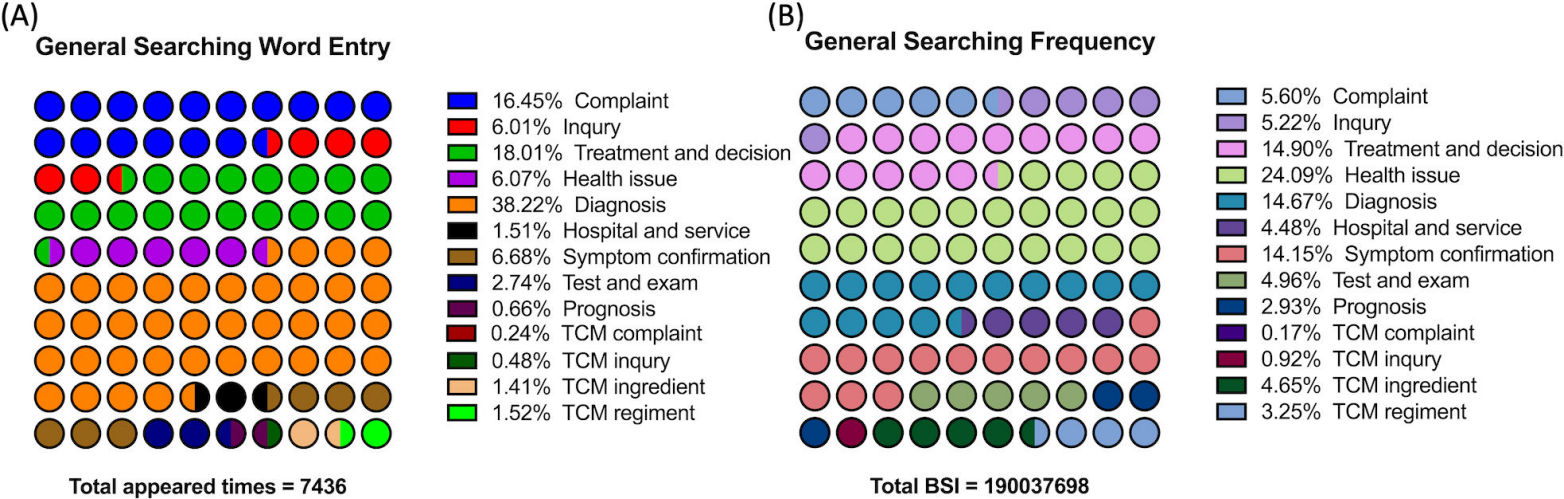
Term categories related to urinary incontinence in the Baidu index demand graph. (A) The distribution of most-appearing related words (word units) that users inquired about in the Baidu index related to urinary incontinence. (B) The distribution of most-searched related words that users inquired about in the Baidu index related to urinary incontinence. BSI: Baidu search index; TCM: traditional Chinese medicine.

During the studied period, 7436 of the 9310 words that appeared were classified as valid user demands related to the topic. The total BSI for these user demand terms was 190,037,698, accounting for only 15.74% (190,037,698/1,207,203,466) of the overall user interest in the topic of UI. Diagnosis-related issues were the most frequent, making up 38.22% (2842/7436) of the total. Treatment- and decision-related issues, as well as complaints, followed. In terms of popularity, health issues received the most attention, accounting for 24.09%. The popularity ratios for the top 3 frequent themes were 14.67%, 14.90%, and 5.60%. This suggests that while UI requests are steady, their value might be overshadowed by other health issues that spike in search popularity. Detailed distributions of these related terms and their search frequencies are depicted in Figure 5. Tables1  2 present the top 3 related terms that were most frequently searched.

**Table 1.** The top 3 most-appearing related words (word units) that users inquired about in the Baidu index related to urinary incontinence.

Category^a^	Term 1	Number of times term appears	Term 2	Number of times term appears	Term 3	Number of times term appears
A	漏尿 (Urinary leakage)	203	尿潴留 (Urinary retention)	112	尿急 (Urinary urgency)	67
B	漏尿是什么原因造成的 (What is the cause for urinary leakage?)	101	尿失禁的原因 (What is the cause for urinary incontinence?)	60	女性漏尿是什么原因 (What is the cause for female incontinence?)	32
C	尿失禁怎么治疗 (How to treat urinary incontinence)	79	凯格尔运动 (Kegel exercise)	50	米多君 (Midodrine)	39
D	盆底肌 (Pelvic muscle)	37	尿道括约肌 (Sphincter urethra)	34	子宫 (Uterus)	14
E	尿失禁 (Urinary incontinence)	421	压力性尿失禁 (Stress incontinence)	258	急迫性尿失禁 (Urge incontinence)	198
F	泌尿外科 (Department of Urology)	4	浙江省中医院 (Zhejiang Provincial Hospital of TCM^b^)	3	北京大学第一医院 (The First Affiliated Hospital of Peking University)	2
G	尿路感染的症状 (Symptoms of urinary tract infection)	33	前列腺增生的症状 (Symptoms of benign prostate hyperplasia)	26	子宫脱垂的症状 (Symptoms of uterus prolapse)	21
H	PSA (Prostate-specific antigen)	5	尿常规能检查出什么 (Clinical indications from urine routine test)	5	膀胱镜 (Cystoscope)	5
I	前列腺炎有什么症状和危害 (Hazards and symptoms of prostatitis)	21	尿酸高有什么严重后果 (Severe consequence of hyperuricemia)	2	憋尿的危害 (Hazard of pee holding)	2
J	伤寒 (Febrile disease)	2	舌苔黄腻 (Yellow and greasy tongue fur)	2	女性肾虚 (”Shen” essence insufficiency of ”Yin” in female)	1
K	肾虚的表现症状有哪些 (Top 10 manifestations of ”Shen” essence insufficiency)	4	中医养生项目 (Body maintenance project of TCM)	1	中医怎么减肥 (How to lose weight with TCM)	1
L	枇杷叶煮水的功效与作用 (Efficacy of folium eriobotryae decoct)	6	五味子的功效与作用 (Efficacy of *Schisandra chinensis*)	4	南瓜子的作用与功效 (Efficacy of cushaw seed)	3
M	补中益气丸的功效和作用 (Efficacy of “BuZhongYiQiWan” pill)	9	金匮肾气丸 (“JinKuiShenQiWan” pill)	9	金匮肾气丸的作用与功效 (Efficacy of “JinKuiShenQiWan”)	5

a A: complaint; B: etiology and causes; C: treatment and pharmaceutical; D: health care–related terms; E: diagnosis; F: health care services and commodities; G: diagnosis confirmation; H: test and exam; I: prognosis; J: TCM diagnosis; K: TCM diagnosis confirmation; L: TCM regimen; M: TCM remedy and materials.

b TCM: traditional Chinese medicine.

**Table 2.** The top 3 most searched related words that users inquired about in the Baidu index related to urinary incontinence.

	Term 1	BSI	Term 2	BSI	Term 3	BSI
A	漏尿 (Urinary leakage)	1,317,720	尿潴留 (Urinary leakage)	1,260,048	阴道前壁膨出 (Anterior vaginal wall prolapse)	651,482
B	尿频繁是什么原因 (What are the causes of urinary frequency?)	1,454,404	尿频尿急尿不尽是什么原因造成的 (What are the causes of urinary frequency, urgency, and incomplete voiding?)	1,323,360	漏尿是什么原因造成的 (What are the causes of urinary leakage?)	862,462
C	凯格尔运动 (Kegel exercise)	1,919,912	甲钴胺片的功效与作用 (The efficacy of Mecobalamin tablet)	1,590,308	提肛运动怎么做才正确 (How to do anus lifting [Kegel] correctly)	1,461,760
D	知网 (CKNI.com)	18,351,618	各城市首轮感染高峰期预测 (Prediction of pandemic peak in each city)	3,739,154	新冠感染 5 次必死 (Certain death after 5 times of covid-19 infection)	2,141,998
E	前列腺炎 (Prostatitis)	2,585,588	尿失禁 (Urinary incontinence)	2,257,708	尿路感染 (Urinary tract infection)	1,631,582
F	京东 (JD.com)	7,659,590	好大夫在线 (Haodaifu.com)	92,324	医生 (Doctors)	82,880
G	尿路感染的症状 (What are the symptoms of urinary tract infections?)	4,361,232	尿毒症的早期症状 (What are the early symptoms of uremia?)	2,467,396	糖尿病的症状有哪些 (What are the symptoms of diabetes mellitus?)	1,947,950
H	抗原检测怎么做 (How to do antigen testing?)	3,788,050	核酸检测多久出结果 (How long does it take for nucleic acid testing?)	1,006,772	HPV (HPV [human papillomavirus])	315,724
I	前列腺炎有什么症状和危害性 (What are the hazards and symptoms of prostatitis?)	5,092,960	肛门口摸到个软软的肉要紧嘛 (Is it consequential to notice a palpable perianal mass?)	130,732	肝囊肿是怎么回事?有危险吗 (What is a hepatic cyst, is it dangerous?)	84,436
J	脾虚 (Pi [spleen] insufficiency)	64,268	伤寒 (Typhoid fever)	29,578	肾阳虚 (‘Shen’ essence insufficiency of ‘Yang’)	26,398
K	肾虚的表现症状有哪些 (What are the symptoms of insufficiency in Shen essence?)	608,124	风热感冒和风寒感冒的症状区别 (Symptom differentiation of FengRe [heat wind origin] fever and FengHan [cold wind origin] cold)	433,660	湿热疹怎么治疗能除根 (How to eradicate “humid heat” rashes?)	84,050
L	陈皮的功效与作用 (The efficacy of tangerine peel)	853,524	石斛的功效与作用 (The efficacy of dendrobium)	808,556	黄芪的功效与主治 (The efficacy and indication of using astragalus membranaceus)	790,142
M	补中益气丸的功效和作用 (The efficacy of “BuQiYiZhongWan” pill)	935,942	金匮肾气丸 (“JinKuiShenQiWan” pill)	647,778	六味地黄丸 (“LiuWeiDiHuangWan” pill)	559,834

a A: complaint; B: etiology and causes; C: treatment and pharmaceutical; D: health care–related terms; E: diagnosis; F: health care services and commodities; G: diagnosis confirmation; H: test and exam; I: prognosis; J: traditional Chinese medicine diagnosis; K: traditional Chinese medicine diagnosis confirmation; L: traditional Chinese medicine regimen; M: traditional Chinese medicine remedy and materials.

### Assessment of the Content Quality of the Baidu Encyclopedia

On the Baidu Encyclopedia platform, 40 chapters were identified and titled with UI. There were 32 chapters accessible for content reference, and each of these chapters could be matched to one of the 5-point scale standards of the global quality score system based on their content quality. There were 8 chapters removed from the analysis due to their book-introducing nature. Disparities in the accessibility of the English title and video link were reported. The number of sections, word count, and number of page visits differed significantly (Table 3).

**Table 3.** Characteristics of the urinary incontinence content on Baidu Encyclopedia.

		GQS^a^ 1 (N=13)	GQS 2 (N=7)	GQS 3 (N=4)	GQS 4 (N=3)	GQS 5 (N=5)	*P* value
**English title**							.01^b^
	Yes	5	0	0	0	4	
	No	8	7	4	3	1	
**Video link**							.04^b^
	Yes	1	1	0	1	4	
No	7	6	4	2	1	
Number of sections, median (IQR)		0 (0-0)	2 (2-2.5)	5 (3-7.75)	6 (6-7)	6 (6-7)	<.001^c^
Word count, median (IQR)		105 (50-252)	707 (610-780)	1334 (931.25‐1788.5)	3002 (2110‐3339.5)	1909 (1906-4633)	.002^c^
Number of page visits, median (IQR)		4789 (1228-7592)	27,114 (15,587.5‐68,407)	8203 (6480.25‐20,747)	21,288 (14,095.5‐131,130)	329,725 (127,846-473,125)	<.001^c^

a GQS: global quality score.

b Fisher exact test.

c Kruskal-Wallis test.

## Discussion

### Principal Findings

Using the most popular search engine, Baidu, made it possible to conduct research with the broadest conceivable national scope and depth [21]. With billions of Chinese users submitting requests daily, it is possible to identify and address the care-seeking demand and decision-making conundrum quickly. To our knowledge, this study represents the initial foray into infodemiology research to elucidate patients’ perceptions and awareness of UI from a clinical and health care standpoint, drawing upon data from billions of users [19,26]. By analyzing a comprehensive dataset spanning 13 years at a national level, it is possible to uncover valuable insights into the prevailing trends, topics of interest, user search behavior, disease recognition, and demographic characteristics.

Within UI issues, topics can be created focusing solely on complaints, inquiries, and treatments using current search keywords. Interest in symptom complaints made up 71.25% of the total search volume, involving over 11 UI-related keywords. Inquiries and treatment requests comprised 3 words (14.57%) and 4 words (14.18%), respectively. Despite the popularity of UI complaint searches, it is important to note that there were specific types of UI complaints, such as those related to postpartum, urgency, older adult patients, and gender-specific conditions. This indicates that the Chinese population has a detailed and specific recognition of UI, requiring professional attention in each subfield. For inquiries and treatments, the search keywords were broader, focusing on exploring causes and potential remedies.

There has been less research focused on exploring the prognosis of UI compared to earlier studies on other subjects [21,26,27]. Several variables may underpin this finding. First, compared to other commonly experienced lower urinary tract symptoms such as frequency, urgency, and incomplete urination, UI exhibits a higher degree of predictability and controllability regarding its onset and occurrence. By definition, the most dominant UI types—SUI and mixed urinary incontinence—addressed the feature of “involuntary urine leakage when straining or during exerting efforts” [28,29]. Dealing with a UI is contingent upon and subordinate to a specific preceding action.

Further, in other UI types, the symptoms tended to be overwhelmed by other stronger senses, such as urgency in urgent UI or being mostly asymptomatic as with neurogenic UI or true incontinence [28,29]. Hence, acquiring knowledge about the diagnosis, etiology, and treatment options for UI is essential for addressing the concerns of those affected by this condition. Consequently, medical practitioners must prioritize raising awareness and promoting the recognition of UI prognosis.

In this study, several search keywords were identified as inquiries about etiology and treatment options, but these words were mere variations of a simple open-ended question. Though self-assisted cyber diagnosis predisposing individuals to delay treatment has been a concern for a long time [30], the limited popularity of searching for these 2 topics shows that the subjective treatment goal of UI has yet to be recognized and established [31]. Therefore, local urologists still have the opportunity to fill these shoes.

The search trends for the 3 UI topics showed some similarities with an initial growth phase. The complaint topic maintained an increasing trend for 10 years, with an APC of only 1.7%, but a steady demand due to high initial search volumes and an average annual increase of 0.1%. UI inquiries and treatments were more variable. Inquiries showed sharp growth from 2016, peaking and then gradually declining by 2019. Treatment searches saw a strong increase over 8 years with an APC of 20.3%, but then the APC dropped sharply by 36.9% from 2018 to 2021. Fluctuations in search popularity may be linked to public reactions to controversies and criticisms of Baidu’s marketing strategies [30,32]. However, during the initial COVID-19 outbreak in Wuhan, China, Baidu remained a key source of reliable information [10,18]. Hence, daily searches for UI topics remained steady at 2000 BSI, indicating a stable interest in general UI information.

Considering the geographic distribution, East China led UI searches across all 3 topics, averaging 30% of the total requests over the past 13 years. North China followed, with an average of 17%. The Northwest region consistently had the lowest average, accounting for 7% of total UI requests, which is lower than other regions, which averaged around 10%. The high number of requests from East China and North China aligns with our previous findings on the premature ejaculation issue [21] due to higher economic levels and population density reported by [33-35]. According to Xue et al, the reported UI frequency in the female population ranged from 8.7% to 69.8%, representing approximately 43‐439 million people [4]. Additionally, the prevalence of UI in males ranges from 17.3% to 35%, according to the existing literature [36]. However, it is important to note that the available data may only provide a limited understanding of the overall prevalence of male UI nationally. The focus of medical professionals on this issue is significantly determined by the problematic UI situation revealed in [2], and there is a shortage of comprehensive surveys on this topic. Less than 25% of survey participants sought medical consultation for UI [36]. Therefore, traditional epidemiological methods may not adequately capture the full scope of morbidity among individuals affected by UI, which often remains overlooked as a clinical issue.

UI is a prevalent clinical condition that impacts individuals of both genders. The primary symptoms of UI vary between genders and age groups, as influenced by the underlying mechanisms associated with each clinical subtype [28,29,37]. However, these clinical conditions share similarities and primarily involve involuntary urine leakage, which is not widely recognized by the general public. This is evident in the users’ search behavior, as indicated by the prevalence of related search terms. Our research shows that the majority of UI inquiries come from women, with the proportions of complaints, inquiries, and treatment-related themes being 67.15%, 68.61%, and 59.12%, respectively. The research focus among individuals aged 20‐29 and 30‐39 years primarily centers around urinary system disorders and their underlying mechanisms, comprising about 60% of research efforts within these age groups. Yet, most searches for disease treatment were from those over 50, accounting for 51.79% of all searches. This result suggests that disparities and inequalities in sociodemographic factors are a significant concern. Although relevant domestic research in Mainland China is lacking, a recent population-comparable study found that sociodemographic characteristics such as age, education, family income, and parity may have had a role in the reported occurrence rate of UI [38,39].

In simpler terms, varying levels of education and socioeconomic status affect how people perceive beliefs, tolerate UI symptoms, and seek health care. Additionally, aging exacerbates UI and is widely recognized as a significant risk factor [40]. Moreover, socioeconomic factors greatly influence web-based search behavior on platforms such as Baidu. People from higher socioeconomic backgrounds generally have better internet access and digital literacy skills, enabling them to search for health information more effectively [41,42]. They tend to use precise medical terminology and look for reputable sources and research. Conversely, individuals from lower socioeconomic backgrounds face barriers like limited internet access, lower digital literacy, and less trust in web-based health information [12]. They may rely on less accurate sources, and their search queries might be more symptom-focused than diagnostic. Cultural and societal influences also shape which health issues people are willing to search for and discuss openly. Higher educational attainment often leads to more proactive information-seeking behaviors and a better understanding of complex health issues. In contrast, those with lower education levels may struggle with medical terminology and have difficulty conducting effective searches [42,43].

Further analysis revealed differences in UI preferences and understanding among various groups. The retrieved search keywords indicated gender- and population-specific demands, showing terms related to men, women, postpartum, and geriatric UI issues. The demographic distribution of these specific UI concerns was also highlighted. As expected, female users made the most inquiries across all demographic-specific UI topics, including those concerning men. In terms of age distribution, the largest group of users was aged 30‐39 years, making up 40.09% of the total. This was followed by those aged 20‐29 and 40‐49 years, who accounted for 29.19% and 29.13%, respectively.

Unlike our previous study on premature ejaculation, which is a male issue with concerns coming mainly from men [21], UI concerns and demands come mostly from women. Intuitively, one would expect patients to be most concerned about their conditions. However, the significant efforts and attention from women within the family context have not been fully recognized. Women play a prominent role in family health care decisions, making about 80% of these choices [44]. Therefore, concerns and demands from women, who may be the wives of husbands or daughters of older adult patients, should not be overlooked. As a result, even content related to male UI issues should consider a women-friendly perspective.

Diagnosis-related issues were the most frequent, making up 38.22% of the total. Treatment- and decision-related issues, as well as complaints, followed. In terms of popularity, health issues received the most attention, accounting for 24.09%. The popularity percentages for the top 3 most frequent themes were 14.67%, 14.90%, and 5.60%, respectively. This suggests that while UI requests are steady, their value might be overshadowed by other health issues that spike in search popularity.

Among the most related words, “urinary leakage,” “urinary retention,” and “urinary urgency” were the most common complaints. In terms of search popularity, “anterior vaginal wall prolapse” ranked third among complaint words. Although these terms relate to UI symptoms, it is unclear if they reflect users’ inquiries or complaints about a symptom. Since different types of UI have similar symptoms but different causes, accurate and thorough UI assessments are needed to prevent misdiagnosis and delayed treatment.

Detrusor hyperactivity with impaired contractile function is a UI-mimicking condition [45]. Characterized by involuntary urine leakage and urgency, the primary cause of this condition is a lack of detrusor contractility. The treatment for this condition is fundamentally different from that of other UI phenotypes. According to a prior prevalence survey on UI misdiagnosis, 24% of older adult nursing home residents have SUI misdiagnosed [46]. Although these facilities offer professional health care services, such conditions cannot be confirmed without implementing cystometry or multichannel videourodynamic evaluation. Therefore, a worse situation of more diagnosis delay and mistreatment could occur if the potential patient only seeks out a doctor via the internet based on their perception of symptoms.

In the treatment topic, we noticed the related terms of Kegel exercise and anus lifting (Tables 1 and 2) were most frequently inquired about with the highest popularity. Pelvic floor muscle training is considered the primary treatment option for UI since it has been demonstrated to effectively reduce episodes of urine leakage and enhance quality of life [47]. Kegel exercises are considerably more popular than other therapies [47] due to their adaptability, simplicity of implementation, and low cost. However, the pelvic strengthening exercises could only moderate the weakened pelvic muscle for better stabilization and hence could mostly be effective in lower-grade UI with moderate symptoms or alleviate severe symptoms of a particular phenotype [31,47,48].

Users who search for information on Baidu can find answers to their questions in Baidu’s official encyclopedia [49]. For UI issues, there were 40 pages describing UI issues ranging from general aspects to certain specific treatment options. Though the content quality varies, the “must knows” of UI, such as its phenotypes, etiologic features, and population-specific issues, were included. Although the convenience of an online encyclopedia platform is well-recognized, the quality and dependability have yet to be sufficiently accurate in resolving confusion and providing health advice [49,50]. Similar to the findings of Handler et al [51], we found that the material on the Baidu platform of popular science is characterized by a high level of detail. However, it is essential to note that this information lacks proper categorization and even includes some inaccurate titles. As a result, not only does this situation impose additional difficulties, but it also has the potential to mislead users, with potentially severe repercussions.

Given these considerations, medical professionals should anticipate patients’ needs and identify relevant information. Adjustments in therapeutic sessions, communication, and public education are essential to address patients’ issues effectively.

There are several limitations to this study. Although Baidu is the largest search platform in Mainland China, other search engines and social media platforms are increasingly competing, which means the daily search-based BSI may not capture all online user demands. Additionally, regional differences in user preferences and political restrictions may exclude search queries from local citizens who use international search platforms legally. Furthermore, as a Chinese-based internet service, requests from foreign languages or non-Chinese minority groups are likely to be systematically excluded. Additionally, all data from the Baidu Index platform were presented in a fixed and finalized format, limiting the ability to further manipulate or reorganize the data for more flexible structural customization.

To the best of our knowledge, this study represents the initial exploration of infodemiology to address the issue of assessing public concern in the Chinese-speaking population around UI. We chose to investigate the popularity of UI because of its complex nature and the potential for a lack of interest, which can lead to hesitation and delayed treatment. By examining current trends and demand, we can understand prevailing patterns and identify patients’ main concerns. This insight allows for adjustments to health care strategies and responses to enhance Medicare services.

### Conclusion

Web-based UI searching is continuous and traceable since January 2011. Different categorized themes within the UI topic highlight specific demands from various populations, necessitating tailored responses. Although web-based platforms can offer answers, medical professionals’ involvement is crucial to avoid misdiagnosis and delayed treatment.

## Supplementary material

10.2196/55670 Multimedia Appendix 1 List of keywords used in composite search index.

## Abbreviations

AAPC: average annual percent change
APC: annual percent change
BSI: Baidu search index
SUI: stress urinary incontinence
TCM: traditional Chinese medicine
UI: urinary incontinence
