# Supplementary material for: Internet Health Information–Seeking Trend of Urinary Incontinence in Mainland China: Infodemiology Study
Source: JMIR Form Res. 2025 Jun 23;9:e55670. doi: 10.2196/55670 (PMC12208350; doi:10.2196/55670)
Supplement: Multimedia Appendix 1 [file formative-v9-e55670-s001.docx]

| List of keywords used in composite search index | | |  |
| --- | --- | --- | --- |
| Domain of terms | Available term in Search engine | English equivilent terms | |
| Complaint | 产后尿失禁 | Post-partum urinary incontinence | |
|  | 急迫性尿失禁 | Urgent urinary incontinence | |
|  | 老年人尿失禁 | Urinary incontinence in the elderly | |
|  | 老人尿失禁 | Elderly urinary incontinence | |
|  | 男性尿失禁 | Male urinary incontinence | |
|  | 尿失禁 | Urinary incontinence | |
|  | 女性尿失禁 | Female urinary incontinence | |
|  | 女性压力性尿失禁 | Female stress urinary incontinence | |
|  | 压力性尿失禁 | Stress urinary incontinence | |
|  | 张力性尿失禁 | Strain urinary incontinence | |
|  | 真性尿失禁 | Genuine urinary incontinence | |
| Enquiry | 真性尿失禁的原因 | What are the causes of genuine urinary incontinence | |
|  | 女性漏尿是什么原因 | What are the causes of female urinary leaking | |
|  | 什么是尿失禁 | What is urinary incontinence | |
| Treatment | 尿失禁的治疗方法 | Treatment of urinary incontinence | |
|  | 尿失禁怎么治疗 | What are the symptoms of bladder cancer | |
|  | 尿失禁怎么办 | Bladder cancer symptoms | |
|  | 怎样治疗尿失禁 | Late stage bladder cancer | |
|  | | | |
